# Do Herbal Formulas Influence the International Normalized Ratio of Patients Taking Warfarin? A Retrospective Study

**DOI:** 10.1155/2015/213927

**Published:** 2015-03-10

**Authors:** Hsu-Yuan Lu, Seung-Yeon Cho, Seong-Uk Park, Woo-Sang Jung, Sang-Kwan Moon, Jung-Mi Park, Chang-Nam Ko

**Affiliations:** Department of Cardiology and Neurology, College of Korean Medicine, Kyung Hee University, No. 26 Kyungheedae-ro, Dongdaemun-gu, Seoul 130-872, Republic of Korea

## Abstract

Warfarin is a common anticoagulant agent for cardiovascular diseases, and it is known to interact with several foods and drugs. Several studies report an interaction between warfarin and herbal medicines; however, the influence of herbal medicines on the international normalized ratio (INR) is still controversial. We investigated the influence of herbal formulas on INR of patients taking warfarin. We searched electronic medical records of inpatients for INR results. Then, we compared the changes in INR and any adverse events between the group taking herbal formulas and warfarin (herbal group) and another group taking warfarin only (nonherbal group). Eighty-six patients were included; 45 patients were assigned to the herbal group and 41 patients to the nonherbal group. The herbal group had taken the same dose of warfarin for a longer period. The nonherbal group had a slightly higher mean INR value than the herbal group. The ratio of INR less than 2 and greater than 3, the ratio of INR that increased or decreased by one or more compared to the initial INR, and the ratio of adverse events were not significantly different between the two groups. It is suggested that use of herbal formulas may not influence INR value.

## 1. Introduction

Warfarin is a common anticoagulant agent that is used in various cardiovascular diseases, venous thrombosis, prevention of systematic embolism, atrial fibrillation, and cardiac valve diseases [1]. It is known to interact with several drugs and foods to influence the international normalized ratio (INR) [2]. Herbal formulas have been used for several thousands of years in China, Korea, and Japan; in recent years, many Western countries have started using herbal formulas [3]. As many countries use herbal formulas, the potential effects of these herbal formulas on the INR have been reported [4–6]. In one report, an herbal formula combination of* Tribulus terrestris*,* Avena sativa*, and* Panax ginseng* was reported to result in a sudden increase in the INR of patients who took warfarin for aortic valve replacement or atrial fibrillation [4]. However, another randomized controlled trial study reported that* Panax ginseng* did not have any effect on INR [7]. Whether or not herbal formulas influence INR is still controversial.

Several previous researchers have reported interactions between herbal formulas and warfarin, but there are few citations published in Science Citation Index (SCI) journals, and there are some important limitations in all of the studies [8–15]. Choi et al. investigated the interaction between herbal formulas and warfarin in 27 patients using the World Health Organization-The Uppsala Monitoring Centre causality categories and modified drug interaction probability scale (mDIP scale). However, the study could not clearly clarify interactions between herbal formulas and warfarin because the mDIP scale only measures single components and is not suitable for complex formulas. Kim et al. [9], Lee et al. [10], Kim et al. [11], and Lee and Ryu [12] investigated patients taking warfarin who were hospitalized at a traditional Korean Medicine hospital. Kim et al. [9] reported that herbal formulas may slightly affect INR, but other studies reported that herbal formulas did not affect INR [10–12]. Kwon et al. [13] and Jung et al. [14] investigated the mean INR of patients taking warfarin and found no statistically significant differences between INR before and INR during the use of the herbal formulas. The results by Jung et al. [14] are limited because they only investigated the mean INR; the data does not show the change in INR in the individual subjects as a result of the herbal formulas. In another study, Kwon et al. [15] investigated 28 patients who took* Panax ginseng* in conjunction with warfarin. Because the study only investigated herbal formulas that included* Panax ginseng*, it was difficult to apply the findings to other herbal formulas that do not contain* Panax ginseng*.

In this study, we compared a group taking herbal formulas in conjunction with warfarin (herbal group) and another group taking warfarin only (nonherbal group) in order to investigate whether herbal formulas affect INR.

## 2. Materials and Methods

The study was performed in accordance with the ethical standards of the Helsinki Declaration. The Institutional Review Board of Kyung Hee University Hospital at Gangdong approved this study (KHNMC-OH-IRB 2013–015).

We searched electronic medical records of patients hospitalized from June 2006 to May 2013 at the Department of Korean Medicine or the Department of Physical Medicine and Rehabilitation in Stroke and Neurological Disorders Center, Kyung Hee University Hospital, at Gangdong to find patients who satisfied our criteria: (1) 18 years of age or older, (2) previously taking warfarin before hospitalization and taking the same dose of warfarin for at least five days since the day of hospitalization, (3) initial INR in the range of 2 to 3, and (4) follow-up measurements conducted once or more during hospitalization. In order to exclude the possible effects on INR, any patients that had taken any herbal medicine in the 30 days before hospitalization were excluded. If the patients were hospitalized more than twice, we regarded each hospitalization as an individual subject. In addition, we designated patients who took herbal formulas with warfarin as the herbal group and those who took only warfarin as the nonherbal group. We then investigated the following items for up to 30 days: (1) ratio of INR less than two and greater than three, (2) ratio of INR changed by one or more from the initial INR, (3) mean INR, and (4) ratio and types of adverse events. If warfarin dose was changed, a patient was discharged in either group, or a patient in the nonherbal group started taking an herbal formula, we investigated INR and adverse events up to the time of that change.

Herbal formulas were prescribed decoctions or extracts. Herbal medicines for decoctions were purchased from Kyung Hee Herb Pharm Co. (Seoul, Korea), and extracts were purchased from the following companies: Kyung Hee Herb Pharm Co. (Seoul, Korea), Tsumura Co., Ltd. (Tokyo, Japan), Hamsoa Pharmaceutical Co., Ltd. (Seoul, Korea), Kwang Dong Pharmaceutical Co., Ltd. (Seoul, Korea), Jeil Hanbang Co., Ltd. (Seoul, Korea), Hankook Shinyak Pharm Co., Ltd. (Chungcheongnam-do, Korea), and Kiwha Bio Co., Ltd. (Gyeongsangnam-do, Korea). Decoctions were prepared using two or more herbal ingredients in 1.2 to 1.5 liters of water boiled in decoction vessels at greater than 100°C for 2 hrs 30 min. All patients in the herbal group took herbal decoctions of 50 mL to 120 mL, 2 hrs after every meal, and took extracts of 6–18 g according to symptoms, if needed.

### 2.1. Statistical Analyses

Data are expressed as number or mean ± standard deviation. Categorical variables were analyzed by Pearson's Chi-square test, and continuous variables were analyzed by Student's *t*-test. *P* values less than 0.05 were considered significant. All data were analyzed using SPSS for Windows, version 18.0 (Statistical Package for the Social Sciences Inc., Chicago, IL, USA).

## 3. Results

Eighty-six patients were included; 45 patients were assigned to the herbal group and 41 patients were assigned to the nonherbal group. The herbal group took the same dose of warfarin for a longer period (*P* = 0.027), had more cerebral infarction as an indicator of warfarin use (*P* = 0.036), and showed a higher hemoglobin count (*P* = 0.045) compared to the nonherbal group. There were no statistically significant differences in other baseline characteristics between the two groups (Table 1).

There were no statistically significant differences in the ratio of INR less than 2 or greater than 3 or the ratio of increase/decrease of more than one from the initial INR between the two groups. However, the nonherbal group had a slightly higher mean INR value than the herbal group (Table 2).

No major bleeding was reported in any patients, but there were some minor bleeding events in both groups. In the herbal group, one patient appeared to have hemoptysis during tracheostomy suctioning, one showed hematuria, and one patient showed melena. In the nonherbal group, one patient complained of hematuria, and another patient showed hemoptysis and subconjunctival hemorrhage. None of these adverse events were fatal, and all patients showed recovery. There was no statistically significant difference in the adverse events ratio between the two groups (Table 3).

## 4. Discussion

Warfarin is an antagonist of vitamin K and is known to interact with several foods and drugs [2]. Because of its narrowing therapeutic index, patients require consistent monitoring when taking warfarin [16]. Several studies have reported that herbal formulas affect INR; however, there were limits to the research methods used in these studies, so the issue is still controversial [7–15]. In this study, we found no substantial differences between the herbal and the nonherbal groups with regard to the ratio of INR less than two and greater than three or the ratio of INR value changed by one or more units from the initial INR. Some previous studies reported that herbal medicines could affect INR [17, 18]; in these cases, the herbal medicines were administered by the patients themselves and were not prescribed by experts. In contrast, some randomized controlled trials reported that herbal formulas prescribed by experts did not affect INR [10–14]. Similar results were shown in our study; therefore, we concluded that even if some patients took herbal formulas that contain* Panax ginseng*,* Dong quai*, and other medicines that can affect INR [19], the influence may be prevented when the herbal formulas were prescribed by experts after medical examination. We described the herbal medicines that had been taken by members of the herbal group in Tables 4–6.

There were three patients in the herbal group and two patients in the nonherbal group that showed adverse events. In the herbal group, one patient showed an INR lower than the therapeutic range, a second showed an INR within the normal therapeutic range, and the third showed an INR higher than the therapeutic range. All patients had bleeding risk factors that were directly associated with adverse events such as frequent tracheostomy suctioning, use of Foley catheter, and history of hematochezia. In the nonherbal group, one patient showed a normal therapeutic INR range with bleeding risk factors, such as the use of a Levin tube and a Foley catheter, and the other patient showed a higher INR than the therapeutic range and had no other bleeding risk factors except warfarin. Regardless of INR value or bleeding risk factors, all of these adverse-event cases involved minor bleeding [20], and there was no statistically significant difference in the adverse event ratio between the two groups. Therefore, we concluded that herbal formulas did not increase bleeding tendency when prescribed by experts. However, warfarin can increase bleeding tendency, so continued monitoring is needed.

The difference in mean INR probably occurred because the nonherbal group had taken a higher warfarin dose than the herbal group, although there was no statistically significant difference in the warfarin dose. Furthermore, the mean INR values of both groups were in the therapeutic range [1], indicating that the mean INR values were not clinically meaningful different.

This survey investigated herbal formulas used in clinics for inpatients taking warfarin, so we considered that the clinical situations were fully reflected and all medicines that had been taken were identified. To our knowledge, this is the first retrospective study of a comparison between a warfarin-treated group and a group who took warfarin in conjunction with herbal formulas. We investigated not only mean INR, but also changes in INR and adverse events; therefore, the data will be useful to clinicians and for further studies.

Because our study period was short, we cannot show the long-term effects of herbal formulas on INR, and the data do not represent the influence of herbal formulas on INR that were not within the therapeutic range or the effects of individual herbal medicines. In the future, large-scale, long-term, prospective, and individual herbal medicines studies will be needed.

## 5. Conclusion

In this study, the ratio of INR less than 2 and greater than 3, the ratio of INR that increased or decreased by one or more compared to the initial INR, and the ratio of adverse events were not significantly different between the herbal group and the nonherbal group. It is suggested that concurrent use of prescribed herbal medicine and warfarin may not influence INR value or bleeding tendency compared with conventional therapy.

## Figures and Tables

**Table 1:** Baseline characteristics between the herbal and nonherbal groups.

	Herbal group (*n* = 45)	Nonherbal group (*n* = 41)	*P* value
Male/female	19/26	17/24	0.943
Age (year)	70.0 ± 12.0	70.5 ± 12.8	0.842
Admission days	26.5 ± 5.7	23.9 ± 7.6	0.077
Period of taking the same dose of warfarin (days)	15.6 ± 8.0	11.9 ± 7.6	0.027^*^
Reason for hospitalization			0.274
Cerebral infarction	43	38	
Intracerebral hemorrhage	0	2	
Epidural hemorrhage	0	1	
Subarachnoid hemorrhage	1	0	
Parkinson's disease	1	0	
Medical history			
Hypertension	37	29	0.208
Diabetes mellitus	17	9	0.110
Dyslipidemia	13	16	0.321
Cerebral infarction	10	4	0.118
Indicators of warfarin			
Cerebral infarction	9	2	0.036^*^
Valvular heart disease (non-op)	1	1	0.947
Valvular heart disease (op)	0	3	0.065
Coronary artery disease	0	2	0.134
Atrial fibrillation	35	31	0.812
Pulmonary embolism	1	2	0.503
Patent foramen ovale	1	0	0.337
Aortic dissection	0	1	0.292
Medications			
Antiplatelet drugs	7	10	0.304
Antihypertensive drugs	32	25	0.321
Antidiabetic drugs	13	6	0.112
Antidyslipidemic drugs	14	17	0.318
Warfarin dose (mg/day)	3.0 ± 1.4	3.3 ± 1.5	0.258
Initial INR (INR)	2.4 ± 0.3	2.4 ± 0.3	0.371
Number of tests of INR	4.6 ± 2.5	4.7 ± 2.8	0.981
Initial laboratory findings			
AST (IU/L)	23.8 ± 9.8	23.8 ± 10.3	0.992
ALT (IU/L)	22.8 ± 16.3	24.1 ± 18.9	0.719
BUN (mg/dL)	16.5 ± 6.3	17.6 ± 6.1	0.470
Creatinine (mg/dL)	0.89 ± 0.27	0.86 ± 0.25	0.561
Platelet (×10^3^/*μ*L)	265.9 ± 96.3	254.6 ± 81.2	0.559
Hemoglobin (g/dL)	13.85 ± 5.23	12.12 ± 1.61	0.045^*^
Hematocrit (%)	42.40 ± 31.94	35.78 ± 4.39	0.192

non-op: without operation history, op: with operation history, INR: international normalized ratio, BUN: blood urea nitrogen, AST: aminotransferase, and ALT: alanine transferase.

Student's *t*-test was used for continuous variables, and Pearson's Chi-square test was used for categorical variables. Data are expressed as number or mean ± standard deviation; ^*^ *P* < 0.05 was considered significant.

**Table 2:** Changes in the international normalized ratio between the herbal and nonherbal groups.

	Herbal group (*n* = 45)	Nonherbal group (*n* = 41)	*P* value
INR greater than 3 (%)	9 (20.0)	16 (39.0)	0.052
INR less than 2 (%)	27 (60.0)	17 (41.5)	0.086
INR changed by one or more units (%)	12 (26.7)	11 (28.8)	0.986
INR increased by one or more units (%)	7 (15.6)	6 (14.6)	0.905
INR decreased by one or more units (%)	5 (11.1)	6 (14.6)	0.625
Mean INR (mean ± SD)	2.3 ± 0.4	2.4 ± 0.4	0.032^*^

INR: international normalized ratio.

Data are expressed as number or mean ± standard deviation; ^*^ *P* < 0.05 was considered significant. Statistical analysis by Pearson's Chi-square test.

**Table 3:** Adverse events ratio between the herbal and nonherbal groups.

	Herbal group (*n* = 45)	Nonherbal group (*n* = 41)	*P* value
Patient number (%)	3 (6.7)	2 (4.9)	0.723

Data are expressed as number or mean ± standard deviation; *P* < 0.05 was considered significant. Statistical analysis by Pearson's Chi-square test.

**Table 4:** Frequency of use of herbal medicines taken by patients with an INR less than 2.

Herbal medicines	Frequency (%)
*Glycyrrhiza uralensis* Fisch., *Poria cocos* (Schw.) Wolf	27 (2.1)

*Angelica gigas* Nakai, *Dioscorea batatas* Decne., *Scutellaria baicalensis* Georgi,	26 (1.1)

*Cinnamomum loureirii* Nees, *Zizyphus jujuba* var. *inermis* Rehder, *Ledebouriella divaricata* (Turcz.) Hiroe, *Panax ginseng* C. A. Mey.	24 (1.8)

*Liriope platyphylla* Wang et Tang, *Paeonia obovata* Max., *Atractylodes japonica* Koidz.	23 (1.7)

*Plantago asiatica* L., *Zingiber officinale* Rosc., *Cnidium officinale* Makino	22 (1.7)

*Bupleurum falcatum* Linne	21 (1.6)

*Raphanus sativus* L., *Apis indica* Radoszkowski, *Citrus unshiu* Markovich	20 (1.5)

*Triticum aestivum* L.	19 (1.4)

*Dryobalanops camphora* Colebr., *Prunus sibirica* L., *Rehmannia glutinosa* (Gaertner) Libosch.	18 (1.4)

Aurum, *Moschus moschiferus* L.	17 (1.3)

*Angelica koreanum* (Max.) Kitagawa, *Pinellia ternata* (Thunb.) Breit.	16 (1.2)

*Glycine max* Merr., *Ampelopsis japonica* (Mak.) Makino, *Zizyphus jujuba* Mill, *Equus asinus* L., *Gazella subgutturosa* Guld., *Schizandra chinensis* (Turcz.) Baill., *Bos taurus domesticus* Gmelin, *Typha orientalis* Presl	15 (1.1)

*Polygala tenuifolia* Willd.	14 (1.1)

*Acorus gramineus* Sol. ex Aiton, *Dimocarpus longan* Lour., *Gardenia jasminoides* J. Ellis	13 (1.0)

*Angelica dahurica* Benth. et Hooker f., *Amomum villosum* Lour., *Alisma canaliculatum* All. Br. et Bouche	12 (0.9)

*Rheum palmatum* var. *palmatum*, *Hordeum vulgare* var. *hexastichon* Aschers., *Inula helenium* L., *Cimicifuga japonica* Spreng., *Atractylodes japonica* Koidz., *Cyperus rotundus* L.	11 (0.8)

*Pueraria thunbergiana* Benth., *Chrysanthemum indicum* L., *Citrus aurantium* L.,* Poncirus trifoliata* Rafin., *Astragalus membranaceus* Bunge, *Coptis chinensis* Franch	10 (0.8)

*Heracleum hemsleyanum* Michx, *Ephedra sinica* Stapf., *Crataegus pinnatifida* Bge, *Cornus officinalis* Sieb. et Zucc., *Coix lacryma-jobi* L, *Phellodendron amurense* Rupr., *Magnolia officinalis* Rehder et Wilson, *Forsythia koreana* Nakai	9 (0.7)

*Paeonia suffruticosa* Andrews, *Polygonum multiflorum* Thunb., *Phyllostachys nigra* var. *henonis* (Bean.) Stapf, *Schizonepeta tenuifolia* (Benth.) Briq.	8 (0.6)

*Castanea crenata* S. et Z., *Thuja orientalis* L., *Plantago alata* Nakai, *Asparagus cochinchinensis* Merr., *Aconitum carmichaeli* Debx, *Areca catechu* L.	7 (0.5)

*Trichosanthes kirilowii* Maxim., *Arisaema amurense* Maximowicz, *Cannabis sativa* L., *Asarum sieboldii* var. *seoulense* Nakai, *Perilla frutescens* var. *acuta* Kudo, *Achyranthes bidentata* Bl., *Anthriscus sylvestris* var. *hirtifructus* Hara, *Carthamus tinctorius* L., *Lonicera japonica* Thunb.	6 (0.5)

*Angelica tenuissima* Nakai, *Pogostemon cablin* (Blanco) Benth, *Mentha arvensis* var. *piperascens* Malinv, *Nelumbo nucifera* Gaertner *Polyporus umbellatus* (Pers.) Fries	5 (0.4)

*Juncus effusus* var. *decipiens* Buchen., *Chaenomeles sinensis* Koehne, *Akebia quinata* var. *polyphylla* Nak., *Morus alba* L., gypsum, *Clematis brachyura* Max.	4 (0.3)

*Salvia miltiorrhiza* Bunge., *Sinomenium acutum* Rehder et Wils., *Amomum cardamomum* L., *Curcuma zedoaria* Rosc., *Scirpus fluviatilis* (Torr.) A. Gray, *Panax notoginseng* (Burk) f. H. Chen, *Dendrobium moniliforme* (L.) Sw, *Lamium album* L., *Alpinia oxyphylla* Miq., *Buthus martensii* Karsch, *Anemarrhena asphodeloides* Bunge, *Citrus reticulata* Blanco, Talc	3 (0.2)

*Terminalia chebula* var. *tomentella* Kurt., *Oryza sativa* L., *Dianthus chinensis* L., *Cibotium barometz* J. Smith, *Capreolus capreolus ochracea* Thomas, *Eucommia ulmoides* Oliv., *Vitex trifolia* L., *Sinapis alba* L., *Liquidambar orientalis* Mill., *Styrax benzoin* Dryand., *Acanthopanax seoulense* Nakai,* Rhinoceros unicornis* L., *Lindera strychnifolia* (Sieb. et Zucc.), *Pistacia lentiscus* Joel, *Paeonia obovata* Max., *Eugenia caryophyllata* Thunb., *Aquilaria agallocha* Roxb., *Psoralea corylifolia* L., *Croton tiglium* L., *Fritillaria verticillata* var. *thunbergii* (Miq.) Baker, *Polygonum bellardi* var. *effusum* Meisn, *Piper longum* L., *Scrophularia buergeriana* Miq.	2 (0.2)

*Alpinia officinarum* Hance, *Prunus persica* (L.) Batsch, mirabilite, *Bombyx mori* L., *Dolichos lablab* L., *Rubus schizostylus* Lev., *Adenophora triphylla* var. *hirsuta* Nakai, *Morus alba* L., *Perilla sikokiana* Nakai, *Gentiana jamesii* Hemsl., *Arctium lappa* L., *Prunus ishidoyana* Nakai, *Artemisia scoparia* Waldst. et Kit., *Polygonum multiflorum* Thunb., *Uncaria sinensis* (Oliv.) Havil., *Lycium barbarum* L., *Sanguisorba longifolia* Bertol., *Gentiana macrophylla* Pall., *Trichosanthes kirilowii* Maxim., *Cuscuta chinensis* Lam., *Taraxacum sinicum* Kitag	1 (0.1)

**Table 5:** Frequency of herbal medicines taken by patients with an INR greater than 3.

Herbal medicines	Frequency (%)
*Glycyrrhiza uralensis* Fisch., *Angelica gigas* Nakai, *Zizyphus jujuba* var. *inermis* Rehder, *Atractylodes macrocephala* Koidz, *Panax ginseng* C. A. Mey., *Zingiber officinale* Rosc.	9 (2.3)

*Platycodon grandiflorum* (Jacq.) A. DC., *Liriope platyphylla* Wang et Tang, *Poria cocos* (Schw.) Wolf, *Paeonia lactiflora* Pallas, *Dioscorea batatas* Decne., *Ligusticum chuanxiong* Hort, *Scutellaria baicalensis* Georgi	8 (2.1)

Aurum, *Glycine max* Merr. *Saposhnikovia divaricata* Schiskin, *Ampelopsis japonica* (Mak.) Makino, *Apis mellifera* L., *Moschus moschiferus* L., *Bupleurum falcatum* Linne, *Triticum aestivum* L., * Equus asinus* L., * Gazella subgutturosa* Guld., * Dryobalanops aromatica* Gaertn. f., *Bos taurus domesticus* Gmelin, * Cinnamomum cassia* Blume, * Typha orientalis* Presl, * Prunus armeniaca* L. var. *ansu* Maxim.	7 (1.8)

*Raphanus sativus* var. *hortensis* for. *acanthiformis* Makino, * Schizandra chinensis* (Turcz.) Baill., * Rehmannia glutinosa* (Gaertner) Libosch.	6 (1.5)

*Chrysanthemum morifolium* Ramat., *Pinellia ternata* (Thunb.) Breit., *Angelica dahurica* Benth. et Hooker f., * Zizyphus jujuba* Mill, * Dimocarpus longan* Lour., * Polygala tenuifolia* Willd.	5 (1.3)

*Rheum palmatum* L., *Amomum villosum* Lour., *Acorus gramineus* Sol. ex Aiton, * Gardenia jasminoides* var. *grandiflora* (Lour.) Nakai, *Coptis deltoidea* C. Y. Cheng et Hsiao, * Phellodendron amurense* Rupr.	4 (1.0)

*Pueraria thunbergiana* Benth., *Ostericum koreanum* (Max.) Kitagawa, * Hordeum vulgare* L., * Thuja orientalis* L., * Nelumbo nucifera* Gaertner, *Citrus unshiu* Markovich, * Asparagus cochinchinensis* Merr.	3 (0.8)

*Angelica tenuissima* Nakai, * Trichosanthes kirilowii* Maxim., * Juncus effusus* var. *decipiens* Buchen., * Ephedra sinica* Stapf., * Aucklandia lappa* Decne, * Sinapis alba* L., * Polygonum multiflorum* Thunb., gypsum, *Asarum sieboldii* Miq., * Cimicifuga heracleifolia* Kom., * Coix lacryma-jobi* var. *ma-yuen* (Roman.) Stapf, *Peucedanum decursivum* (Miq.) Maxim.,* Citrus aurantium* L., * Atractylodes lancea* (Thunb.) DC., * Corydalis ternata* Nakai	2 (0.5)

*Castanea crenata* S. et Z., *Dianthus chinensis* L., *Lonicera japonica* Thunb., *Arisaema amurense* Maximowicz, *Angelica pubescens* for. *biserrata* Shan et Yuan., *Cannabis sativa* L., *Chaenomeles sinensis* Koehne, *Paeonia suffruticosa* Andrews, *Akebia quinata* Decne., *Commiphora myrrha* Engl., *Mentha arvensis* var. *piperascens* Malinv., *Sinomenium acutum* Rehder et Wils., *Dolichos lablab* L., *Areca catechu* L., *Crataegus pinnatifida* Bge, *Cornus officinalis* Sieb. et Zucc.,* Morus alba* L.,* Dendrobium loddigesii* Rolfe.,* Perilla frutescens* var. *acuta* Kudo,* Forsythia suspensa* (Thunb.) Vahl, *Lindera strychnifolia* (Sieb. et Zucc.),* Gentiana scabra* Bunge,* Clematis mandshurica* Rupr.,* Lonicera japonica* Thunb.,* Buthus martensii* Karsch,* Anemarrhena asphodeloides* Bunge,* Plantago asiatica* L.,* Aconitum carmichaeli* Debx,* Citrus unshiu* Markovich,* Alisma orientalis* (Sam.) Juzep,* Polygonum aviculare* Linne,* Cyperus rotundus* L.,* Scrophularia buergeriana* Miq.,* Schizonepeta tenuifolia* (Benth.) Briq.,* Carthamus tinctorius* L., Talc,* Magnolia officinalis* Rehder et Wilson	1 (0.3)

**Table 6:** Frequency of use of herbal medicines taken by the herbal group.

Herbal medicine	Frequency (%)
*Glycyrrhiza glabra* L.	45 (2.0)

*Angelica acutiloba* Kitag., *Dioscorea tenuipes* Fr. et Sav., *Scutellaria baicalensis* Georgi	49 (1.9)

*Thuja orientalis* L., *Paeonia lactiflora* Pallas, *Poria cocos* (Schw.) Wolf	41 (1.8)

*Liriope platyphylla* Wang et Tang, *Atractylodes macrocephala* Koidz, *Ligusticum wallichii* var. *officinale* Yook, *Zingiber officinale* Rosc.	40 (1.8)

*Platycodon grandiflorum* (Jacq.) A. DC., *Zizyphus jujuba* var. *inermis* Rehder, *Ledebouriella divaricata* (Turcz.) Hiroe, *Cinnamomum cassia* Blume	39 (1.7)

*Panax ginseng* C. A. Mey.	38 (1.7)

*Bupleurum falcatum* Linne	36 (1.6)

*Triticum aestivum* L.	35 (1.5)

*Apis indica* Radoszkowski	34 (1.5)

*Dryobalanops camphora* Colebr.	33 (1.5)

*Raphanus sativus* L., *Prunus sibirica* L.	32 (1.4)

Aurum, *Moschus moschiferus* L., *Rehmannia glutinosa* (Gaertner) Libosch.	31 (1.4)

*Citrus unshiu* Markovich	29 (1.3)

*Glycine max* Merr., *Pinellia pedatisecta* Schott, *Ampelopsis japonica* (Mak.) Makino, *Equus asinus* L., *Gazella subgutturosa* Guld., *Bos taurus domesticus* Gmelin, *Typha orientalis* Presl	28 (1.1)

*Zizyphus jujuba* Mill, *Schizandra chinensis* (Turcz.) Baill., *Polygala sibirica* L.	25 (1.1)

*Notopterygium forbesii* Boiss, *Rheum palmatum* L., *Dimocarpus longan* Lour., *Gardenia jasminoides* var. *grandiflora* (Lour.) Nakai	23 (1.0)

*Acorus gramineus* Sol. ex Aiton, *Coptis chinensis* Franch	21 (0.9)

*Hordeum vulgare* L., *Inula helenium* L., *Amomum villosum* Lour., *Astragalus membranaceus* Bunge	20 (0.9)

*Chrysanthemum indicum* L., *Alisma orientalis* (Sam.) Juzep,* Cyperus rotundus* L., *Phellodendron amurense* Rupr.	19 (0.8)

*Pueraria thunbergiana* Benth., *Cimicifuga heracleifolia* Kom.	18 (0.8)

*Atractylodes lancea* (Thunb.) DC.	17 (0.7)

*Angelica pubescens* for. *biserrata* Shan et Yuan., *Crataegus pinnatifida* Bge, *Citrus aurantium* L., *Magnolia officinalis* Rehder et Wilson	15 (0.7)

*Ephedra sinica* Stapf., *Cornus officinalis* Sieb. et Zucc., *Lonicera japonica* Thunb.	14 (0.6)

*Paeonia suffruticosa* Andrews, *Phyllostachys nigra* var. *henonis* (Bean.) Stapf, *Poncirus trifoliata* Rafin., *Plantago alata* Nakai, *Schizonepeta tenuifolia* (Benth.) Briq.	13 (0.6)

*Angelica tenuissima* Nakai, *Asarum sieboldii* Miq., *Coix lacryma-jobi* L., *Asparagus cochinchinensis* Merr.	12 (0.5)

*Aconitum koreanum* R. Raymond, *Polygonum multiflorum* Thunb., *Nelumbo nucifera* Gaertner	11 (0.5)

*Mentha arvensis* var. *piperascens* Malinv.	10 (0.4)

*Agastache rugosa* (Fisch. et Meyer) O. Kuntze, *Trichosanthes kirilowii* Maxim., *Arisaema erubescens* (Wall). Schott, *Juncus effusus* var. *decipiens* Buchen., gypsum, *Forsythia suspensa* (Thunb.) Vahl, *Achyranthes bidentata* Bl., *Peucedanum decursivum* (Miq.) Maxim., *Areca catechu* L.	9 (0.4)

*Castanea crenata* S. et Z., *Akebia quinata* Decne., *Perilla frutescens* var. *acuta* Kudo	8 (0.4)

*Cannabis sativa* L., *Chaenomeles sinensis* Koehne, *Aconitum carmichaeli* Debx, *Clematis mandshurica* Rupr., *Polyporus umbellatus* (Pers.) Fries, *Anemarrhena asphodeloides* Bunge, *Carthamus tinctorius* L., Talc	7 (0.3)

*Dianthus chinensis* L., *Polygonum aviculare* Linne	6 (0.3)

*Vitex rotundifolia* L. Fil., *Amomum kravanh* Pierre ex Gagnep, *Curcuma zedoaria* Rosc., *Scirpus fluviatilis* (Torr.) A. Gray, *Morus alba* L., *Dipsacus asper* Wall, *Alpinia oxyphylla* Miq., *Citrus unshiu* Markovich	5 (0.2)

*Terminalia chebula* var. *tomentella* Kurt., *Salvia miltiorrhiza* Bunge., *Eucommia ulmoides* Oliv., *Sinomenium acutum* Rehder et Wils., *Panax notoginseng* (Burk.) f. H. Chen, *Dendrobium loddigesii* Rolfe., *Liquidambar orientalis* Mill., *Styrax benzoin* Dryand., *Rhinoceros unicornis* L., *Lindera strychnifolia, Syzygium aromaticum* Merr et Perry, *Aconitum carmichaeli* Debx, *Aconitum ciliare* DC., *Aquilaria agallocha* Roxb., *Piper longum* L.	4 (0.2)

*Cibotium barometz* J. Smith, *Sinapis alba* L., *Acanthopanax sessiliflorus, Psoralea corylifolia* L., *Scrophularia buergeriana* Miq.	3 (0.1)

*Oryza sativa* L., *Cervus nippon* Temminck, mirabilite, *Dolichos lablab* L., *Morus alba* L., *Perilla frutescens* var. *acuta* Kudo, *Gentiana scabra* Bunge,* Arctium lappa* L., *Prunus humilis* Bunge, *Lycium chinense* Mill., *Gentiana macrophylla* Pall., *Cuscuta chinensis* Lam., *Croton tiglium* L., *Fritillaria thunbergii* Miq., *Taraxacum platycarpum* H. Dahlsi, *Corydalis ternata* Nakai	2 (0.1)

*Alpinia officinarum* Hance, *Sophora flavescens* Ait., *Lycium chinense* Mill., *Prunus persica* (L.) Batsch, *Commiphora myrrha* Engl., *Bombyx mori* L., *Tribulus terrestris* L., *Rubus coreanus* Miq., *Spirodela polyrhiza* (L.) Schleid., *Adenophora triphylla* var. *japonica* Hara, *Cryptotympana pustulata* Fabricius, *Prunus mume* Sieb. et Zucc., *Myristica fragrans* Houtt., *Artemisia capillaris* Thunb., *Uncaria sinensis* (Oliv.) Havil., *Gastrodia elata* Bl., *Trichosanthes kirilowii* Maxim., *Amomum tsaoko* Crevost et Lemaire, *Alpinia katsumadai* Hayata, *Sesamum indicum* L., *Erodium stephanianum* Willd.	1 (0.0)
